# Evidence-informed recommendations for constructing and disseminating messages supplementing the new Canadian Physical Activity Guidelines

**DOI:** 10.1186/1471-2458-13-419

**Published:** 2013-05-01

**Authors:** Amy E Latimer-Cheung, Ryan E Rhodes, Michelle E Kho, Jennifer R Tomasone, Heather L Gainforth, Kristina Kowalski, Gabriella Nasuti, Marie-Josée Perrier, Mary Duggan

**Affiliations:** 1 School of Kinesiology and Health Studies, Queen’s University, 28 Division St, Kingston, ON, K7L 3N6, Canada; 2 Behavioural Medicine Laboratory, School of Exercise Science, Physical and Health Education, University of Victoria, STN CSC, PO Box 3010, Victoria, BC, V8W 3N4, Canada; 3 School of Rehabilitation Science, McMaster University, Hamilton, ON, L8S 4K1, Canada; 4 Department of Physical Medicine and Rehabilitation, Johns Hopkins University, Phipps 197, Baltimore, MD, 21287, USA; 5 Department of Kinesiology, McMaster University, 1280 Main St. W, Hamilton, ON, L8S 4K1, Canada; 6 Canadian Society for Exercise Physiology, 18 Louisa St., Suite 370, Ottawa, ON, K1R 6Y6, Canada

**Keywords:** Physical activity guidelines, AGREE II, Messaging, Knowledge translation

## Abstract

**Background:**

Few validated guidelines exist for developing messages in health promotion practice. In clinical practice, the Appraisal of Guidelines, Research, and Evaluation II (AGREE II) Instrument is the international gold standard for guideline assessment, development, and reporting. In a case study format, this paper describes the application of the AGREE II principles to guide the development of health promotion guidelines for constructing messages to supplement the new Canadian Physical Activity Guidelines (CPAG) released in 2011.

**Methods:**

The AGREE II items were modified to suit the objectives of developing messages that (1) clarify key components of the new CPAG and (2) motivate Canadians to meet the CPAG. The adapted AGREE II Instrument was used as a systematic guide for the recommendation development process. Over a two-day meeting, five workgroups (one for each CPAG – child, youth, adult, older adult – and one overarching group) of five to six experts (including behavior change, messaging, and exercise physiology researchers, key stakeholders, and end users) reviewed and discussed evidence for creating and targeting messages to supplement the new CPAG. Recommendations were summarized and reviewed by workgroup experts. The recommendations were pilot tested among end users and then finalized by the workgroup.

**Results:**

The AGREE II was a useful tool in guiding the development of evidence-based specific recommendations for constructing and disseminating messages that supplement and increase awareness of the new CPAG (child, youth, adults, and older adults). The process also led to the development of sample messages and provision of a rationale alongside the recommendations.

**Conclusions:**

To our knowledge, these are the first set of evidence-informed recommendations for constructing and disseminating messages supplementing physical activity guidelines. This project also represents the first application of international standards for guideline development (i.e., AGREE II) to the creation of practical recommendations specifically aimed to inform health promotion and public health practice. The messaging recommendations have the potential to increase the public health impact of evidence-based guidelines.

## Background

Public health practice is often criticized for a lack of evidence-based decision-making in program development and implementation in the area of health behavior change [1]. Indeed, over the past decade, a substantial body of research examining strategies for changing individuals’ health behavior (c.f., [2,3]) has accumulated largely in the absence of systematic processes for disseminating the research findings to public health practitioners. In other health-related fields, evidence-based practice guidelines are a central mechanism for knowledge translation and a foundation for evidence-based decision-making. Evidence-based practice guidelines consolidate research evidence, can be disseminated broadly, and inform decision-making [4,5]. In turn, guideline implementation can improve patient outcomes [6,7]. To address the lack of evidence-based health behavior change practice recommendations, a systematic process for recommendation development is needed. Having an established process has potential to facilitate the translation of health behavior change research into practice and to address a significant research-to-practice gap. The development of recommendations will add practical value to the accumulating body of behavior change research and will create standards of practice with potential to improve the impact of health promotion programs.

In clinical medicine (e.g., oncology, cardiology), the Appraisal of Guidelines, Research, and Evaluation II (AGREE II) Instrument is the international gold standard for guideline assessment, development, and reporting [8]. Encompassing 23 items representing six quality domains (i.e., scope and purpose, stakeholder involvement, rigour of development, clarity of presentation, applicability, editorial independence), the AGREE II Instrument is generic and has potential to be applied to establishing guidelines for any step in the health care continuum (i.e., from health promotion to public health to interventions) [8]. Despite this potential, to our knowledge, the AGREE II has not been applied to guide the use of evidence to inform the development of health behavior change practice recommendations that can be used by public health practitioners. Using a case study format, in this paper we describe a process guided by a modified version of AGREE II for developing evidence-based recommendations for constructing behavior change messages to accompany the Canadian Physical Activity Guidelines (CPAG) for children, youth, adults, and older adults.

### The context

Several countries (e.g., United States, Canada, United Kingdom) and international organizations (e.g., World Health Organization) have recently released new or updated physical activity guidelines [9-13]. These clinical practice guidelines were created through rigorous systematic review and development processes. (c.f., [10]). The CPAG in particular were developed in accordance with the AGREE II [8]. As a whole, these guidelines reflect the most up-to-date evidence for the frequency, intensity, duration and type of physical activity necessary to achieve health benefits. The guidelines do not, however, provide specific information aimed to persuade people to become more active. Thus, to motivate people to become active, the guidelines must be supplemented with messages that convey *how* to achieve the recommended activity level and *why* this is relevant to them (i.e., motivational messages). The responsibility for delivering these motivational messages largely falls to stakeholder organizations and practitioners disseminating the guidelines through their physical activity promotion initiatives (e.g., ad campaigns, brochures, websites). Thus to optimize uptake of the guidelines, organizations and practitioners need to be informed about the new guidelines, and given strategies for constructing motivational messages. To address this need among Canadian organizations, we undertook a two-phase process for developing recommendations for constructing messages to supplement the new CPAG specifically. In Phase 1, a systematic process was used to review the relevant literature. In Phase 2, an expert panel convened to develop the practical recommendations for the constructing messages to supplement the CPAG. The purpose of this case study is to provide an overview of these processes and a model for developing practice recommendations in other health behaviour change domains.

### Phase 1: literature review and background rationale

A systematic process to inform the development of messages supplementing the CPAG was undertaken by the Canadian Society for Exercise Physiology (CSEP). Initially, the CSEP received funding from the Public Health Agency of Canada (PHAC) and the First Nations and Inuit Health Branch of Health Canada to support an evidence-informed review of the physical activity and messaging literature [14]. With additional funding from PHAC, the CSEP commissioned two systematic reviews of specific messaging and behaviour change techniques [15,16]. The major conclusions from these three reviews were as follows:

● Brawley and Latimer [14] recommended that: a) messages should be informative, thought-provoking, clear, and persuasive while targeting constructs from behavior change theories (i.e., self-efficacy) and b) moderators that influence message impact should be identified and considered when creating messages and messaging interventions.

● Latimer, Brawley, and Bassett [15] concluded that although additional research is needed, tailored, gain-framed, and self-efficacy messages seemed to be promising strategies for constructing physical activity messages.

● Rhodes and Pfaeffi [16] identified notable gaps in the physical activity intervention literature and suggested that when developing messages supplementing the guidelines, there may be benefit in emphasizing aspects of self-regulation, self-efficacy and affective outcome expectancies.

Taken together, these three reviews provided the foundational principles for developing the message construction recommendations (Phase 2).

### Phase 2: recommendation development

**Figure 1:**
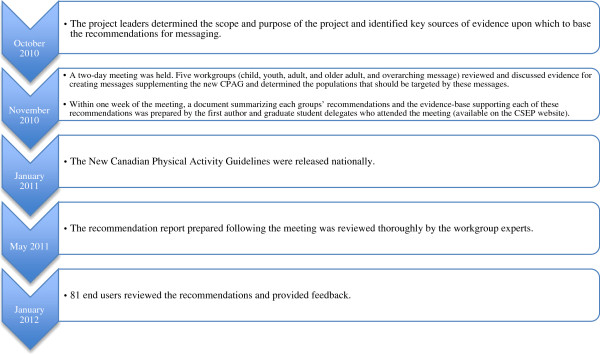
**Timeline for developing and revising the messaging recommendations.** This flow chart outlines the timeline for developing the messaging recommendations.

In Phase 2 of the message development process, an expert panel was convened. The panel was responsible for interpreting the evidence from Phase 1 and developing practical recommendations for constructing and disseminating messages supplementing the new CPAG. We followed a systematic protocol modified from the AGREE II Instrument [8] to ensure rigor, comprehensiveness and transparency in the process of developing the recommendations. The timeline for the project is outlined in Figure 1. The development process was conducted within the mandate of the CSEP program activities. Reporting upon the process including stakeholder evaluations represents secondary use of anonymous information. In accordance with the Tri-Council Policy statement these activities do not fall in the scope of research ethics review.

## Methods

The two lead authors of this paper consulted one of the developers of the AGREE II Instrument to gain an understanding of: a) the methodological underpinnings of each of the 23 AGREE II items and b) how AGREE II principles applied to the objective of developing evidence-based messaging recommendations. With this knowledge, we modified the tool by tailoring the item wording to suit our objective. The modified items are described in Additional file 1. Specifically, we shifted the focus in the original AGREE II from patient populations to the guidelines’ target audiences (children, youth, adults and older adults). We also used a modified evidence review process to accommodate project constraints. When considering the implications of the recommendations, the impact on health outcomes was not considered directly but rather the impact of the recommendations on resource demands for organizations and stakeholders applying the recommendations was considered. The subsequent sections outline the application of the modified AGREE II domains.

### AGREE domain 1: the scope and purpose

The objectives and the practical questions addressed by the recommendations were determined initially by the project leaders in consultation with three key stakeholders – CSEP, ParticipACTION, and PHAC. These three organizations were involved in defining the scope and purpose because they are considered leaders in physical activity promotion within Canada and were slated to jointly disseminate the CPAG. The objectives and practical questions addressed by our recommendations are listed in Additional file 1.

The expert workgroups determined the target populations for each of the messages and recommendations specific to each CPAG. The target populations were determined by reviewing evidence of key social influences on physical activity behavior (c.f. the list of resources included in Additional file 2). The target populations and rationale for these populations are outlined in Tables 1, 2, 3, 4, 5, 6 and the Additional files 3, 4, 5, 6, 7.

**Table 1:** Introductory preamble

	
	**General preamble**
**Content**	The Canadian Society for Exercise Physiology released the new Canadian Physical Activity Guidelines for children, youth, adults and older adults in January 2011. These guidelines reflect the most up-to-date evidence for the frequency, intensity, duration and type of physical activity necessary to achieve health benefits.
	We recognize that guidelines should be supplemented with a comprehensive intervention strategy to help Canadians reach these guidelines. This comprehensive strategy should focus on changing Canadians’ behaviour, the environments in which Canadians live, work, and play, and the policies that support physical activity. The Messaging Recommendations included here have a very specific objective of providing public health practitioners with general principles for constructing messages that a) clarify what is new about the guidelines and b) motivate Canadians to meet the guidelines. The recommendations were developed by a panel of experts including researchers with expertise in promoting physical activity and practitioners with expertise in social marketing and public health. All recommendations are based on current research evidence.
	These recommendations should be used as a starting point for creating messages included in campaigns and information included in resources promoting physical activity. The recommendations will help practitioners identify whom messages should be targeted towards, general content for the messages, and methods for disseminating the guidelines. Because these guidelines are meant to be a series of general principles applicable to a variety of practices and contexts, specific examples and statistics have not been provided. Rather, practitioners will be required to seek information specific their audience. Recommended sources for this information include:
**Recommended Resources**	Physical Activity Guidelines
	● See the guidelines section on the CSEP website: http://www.csep.ca
	Physical activity participation rates and barriers to participation
	● Visit the Canadian Fitness and Lifestyle Research Institute: http://www.cflri.ca
	Benefits of physical activity
	● Refer to the systematic reviews of research evidence informing the development of the new Physical Activity Guidelines http://www.ijbnpa.org/series/canada_physical_activity
	Definitions of key terms
	● See the glossary of terms on the CSEP website: http://www.csep.ca
	Specific examples of strategies to meet the physical activity guidelines
	● Visit the ParticipACTION website: http://www.participaction.com

**Table 2:** General messaging recommendations for the new Canadian Physical Activity Guidelines – overarching messages

	
**Preamble**	
These recommendations are relevant to all practitioners and organizations communicating the new Canadian Physical Activity Guidelines for Children, Youth, Adults, and Older Adults. These recommendations should be used to create awareness and a clear understanding of the new Canadian Physical Activity Guidelines among general population, practitioners, and policy makers.	
**Recommendations**	
**General Awareness**	Overarching awareness messages should inform Canadians that there are new guidelines based on scientific evidence and convey that rigorous scientific reviews have revealed new information about the health benefits of physical activity.
**General Clarification**	Overarching clarification messages should explain why the guidelines have changed and clarify terminology included in the guidelines.

Key References

Janssen, I. & LeBlanc, A. G. (2010). Systematic review of the health benefits of physical activity and fitness in school-aged children and youth. *International Journal of Behavioral Nutrition and Physical Activity, 7*, 40.

Latimer, A. E., Brawley, L. R., & Bassett, R. L. (2010). A systematic review of three approaches for constructing physical activity messages: What messages work and what improvements are needed? *International Journal of Behavioral Nutrition and Physical Activity, 7,* 36.

Paterson, D. H. & Warburton, D. E. R. (2010). Physical activity and functional limitations in older adults: A systematic review related to Canada's Physical Activity Guidelines. *International Journal of Behavioral Nutrition and Physical Activity*,7, 39.

**Table 3:** Messaging recommendations for the new Canadian Physical activity Guidelines for Children

	
**Preamble**	
These recommendations are relevant to all practitioners and organizations communicating the new Canadian Physical Activity Guidelines for Children. These recommendations should be used to create a clear understanding of the new Canadian Physical Activity Guidelines for Children and to construct and to deliver messages that promote achievement of the guidelines.	
**Recommendations**	
**Target Audience**	Messages should target teachers, parents, and children.
**Clarification Messages**	It is imperative for parents and teachers to understand exactly what the guidelines are and that their role is pivotal in the physical activity that children engage in. Through teachers and parents, children will then understand, learn, and potentially make a habit of being active for a minimum of 60 minutes every day, and incorporating muscle and bone strengthening activities at least three times in a week.
**Motivational Messages**	Motivational messages for teachers should encourage them to act as role models, promoting physical activity throughout the school day.
	Motivational messages for parents should reinforce: a) parents’ pivotal role in shaping their child’s interests and attitudes, b) that their support is positively associated with their child’s physical activity, and c) the importance of planning to be physically active with the family.
	Motivational messages for children should be fun, cool, and socially appealing, and may benefit from targeting children’s confidence to engage in physical activity.
	*Examples*
	Invest in your child’s future by planning for physical activity now. Get Active. It’s Fun!
	Being active is as easy as a hop, skip, and a jump. You can do it.
**Channels of Delivery**	Messages should be disseminated to teachers through worksheets that provide practical resources on how to increase physical activity throughout the school day.
	Messages should be disseminated to parents through mass media.
	Messages should be disseminated to children through nongovernmental organizations (e.g., Boys and Girls’ Clubs) and mass media

Key References

Active Healthy Kids Canada website. (2009). 2009 Report Card. http://www.activehealthykids.ca

Active Healthy Kids Canada website. (2011). 2011 Report Card. http://www.activehealthykids.ca

Craig, C., Bauman, A., Gauvin, L., Robertson, J., & Murumets, K. (2009). ParticipACTION: A mass media campaign targeting parents of inactive children: Knowledge, saliency, and trialing behaviors. *International Journal of Behavioral Nutrition and Physical Activity, 6*(1), 88.

Dobbins, M., De Corby, K., Robeson, P., Husson, H., & Tirilis, D. (2009). School-based physical activity programs for promoting physical activity and fitness in children and adolescents aged 6-18. *Cochrane Database of Systematic Reviews* (1), CD007651.

Huhman, M., Potter, L. D., Wong, F.L., Banspach, S.W., Duke, J.C., and Heitzler, C.D. (2005). Effects of a mass media campaign to increase physical activity among children: Year-1 results of the VERB campaign*. Pediatrics, 116*(2), e277-e284.

Lubans, D. R., Foster, C., & Biddle, S. J. H. (2008). A review of mediators of behavior in interventions to promote physical activity among children and adolescents. *Preventive Medicine, 47*(5), 463-470.

Naylor, P. J., Macdonald, H. A., Zebedee, J. A., Reed, K. E. and McKay, H. A. (2006). Lessons learned from Action Schools! BC – An “active school” model to promote physical activity in elementary schools. *Journal of Science and Medicine in Sport, 9*, 413-423.

Oates, C., Blades, M., and Gunter, B. (2003). Marketing to children. Journal of Marketing Management, 19(3-4), 401-409.

Price, S. M., Huhman, M., & Potter, L. D. (2008). Influencing the parents of children aged 9-13 years: Findings from the VERB campaign. *American Journal of Preventive Medicine, 34*(6 Suppl), S267-274.

Rhodes, R. E., Naylor, P.J., and McKay, H.A. (2010). Pilot study of a family physical activity planning intervention among parents and their children. *Journal of Behavioral Medicine, 33*(2), 91-100.

Trudeau, F. and Shepherd, R. J. (2005). Contribution of school programmes to physical activity levels and attitudes in children and adults. *Sports Medicine, 35*(2), 89-105.

Van Der Horst, K., Paw, M.J.C.A., Twisk, J.W.R., Van Mechelen, W. (2007). A brief review on correlates of physical activity and sedentariness in youth. *Medicine & Science in Sports & Exercise, 39*(8), 1241-1250.

Welk, G. J., Wood, K., and Morss, G. (2003). Parental influences on physical activity in children: An exploration of potential mechanisms*. Pediatric Exercise Science, 15*, 19-33.

Zecevic, C. A., Tremblay, L., Lovsin, T., and Lariviere, M. (2010). Parental influence on young children’s physical activity. *International Journal of Pediatrics*, *2010*: 468526.

**Table 4:** Messaging recommendations for the new Canadian Physical activity Guidelines for Youth

	
**Preamble**	
These recommendations are relevant to all practitioners and organizations communicating the new Canadian Physical Activity Guidelines for Youth. These recommendations should be used to create a clear understanding of the new Canadian Physical Activity Guidelines for Youth and to construct and to deliver messages that promote achievement of the guidelines.	
**Recommendations**	
**Target Audience**	Messages should separately target younger youth (12-14 yrs) and their parents and older youth (15-17 yrs) and their parents.
**Clarification Messages**	Clarification messages should: a) provide concise and clear descriptions of the guidelines and physical activity, b) define different levels of physical activity intensity (e.g., vigorous, vs. moderate), c) identify physical activity opportunities, d) include information about where to access more information about physical activity, e) indicate that science has evolved since the release of the old guidelines and that the new guidelines reflect latest evidence about physical activity, f) address the semantic differences between the old and new guidelines (e.g., up to 90 minutes vs. at least 60 minutes), and g) provide a wide variety of examples of physical activity – from active living to sport.
**Motivational Messages**	Motivational messages should emphasize a) parents’ pivotal role in encouraging and supporting their youth’s physical activity, b) a variety of benefits that physical activity offers including emotional, developmental, physical and academic benefits, c) that physical activity can be a collective-social movement that promotes community, active living and the environment, d) the importance of planning physical activity by parents and youth, e) strategies for overcoming barriers to physical activity, and f) empowering youth to choose how they will meet the guidelines.
	*Examples*
	60 minutes every day! As a parent, you can help your teen get there.
	Move your teen to move more.
	Life looks better with physical activity.
	Pick a time. Pick a place. Move your teens to move more.
	Pick a time. Pick a place. How you want to get active is up to you!
	Plan ahead for roadblocks!
	Expand your horizons! How you want to get active is up to you!
**Channels of Delivery**	Messages should be disseminated to parents through websites, mass media campaigns featuring celebrities, magazines and news articles, and smart phone applications.
	Messages should be disseminated to youth through websites, promotional materials within their school, public service announcements featuring celebrities, social media, smart phone applications, and community organizations and events.

Key References

Marcus, B., Owen, N., Forsyth, L., Cavill, N., & Fridinger, F. (1998). Physical Activity Interventions Using Mass Media, Print Media, and Information Technology. *American Journal of Preventive Medicine, 15*(4), 362-278.

Rhodes, R., & Pfaeffli, L. (2010). Mediators of physical activity behavior change among adult non-clinical populations: A review update. *International Journal of Behavioral Nutrition and Physical Activity, 7*(1), 37.

Sallis, J., Prochaska, J., & Taylor, W. (2000). A review of correlates of physical activity of children and adolescents. *Medicine and Science in Sports and Exercise, 32*(5), 963-975.

Strong, W. B., Malina, R. M., Blimkie, C. J., Daniels, S. R., Dishman, R. K., Gutin, B., . . .Trudeau, F. (2005). Evidence based physical activity for school-age youth. *The Journal of Pediatrics, 146*(6), 732-737. doi: http://10.1016/j.jpeds.2005.01.055

Trudeau, F., & Shephard, R. (2008). Physical education, school physical activity, school sports and academic performance. *International Journal of Behavioral Nutrition and Physical Activity, 5*(10). doi: http://10.1186/14795868-5-10

Williams, D., Anderson, E., & Winett, R. (2005). A review of the outcome expectancy construct in physical activity research. *Annals of Behavioral Medicine, 29*(9), 70-79.

Wong, F., Huhman, M., Asbury, L., Bretthauer-Mueller, R., McCarthy, S., Londe, P., et al. (2004). VERB™—a social marketing campaign to increase physical activity among youth. *Preventing Chronic Disease, 1*(3).

**Table 5:** Messaging recommendations for the new Canadian Physical activity Guidelines for Adults

	
**Preamble**	
These recommendations are relevant to all practitioners and organizations communicating the new Canadian Physical Activity Guidelines for Adults. These recommendations should be used to create a clear understanding of the new Canadian Physical Activity Guidelines for Adults and to construct and to deliver messages that promote achievement of the guidelines.	
**Recommendations**	
**Target Audience**	Messages should target inactive adults who do not enjoy engaging in physical activity or recognize that being active can make you feel good.
**Clarification Messages**	Clarification messages should a) emphasize that 150 minutes is the minimum for health benefits as opposed to weight loss, b) clearly describe what is meant by strength versus aerobic training and provide examples of each, c) clearly describe and provide examples of what is meant by moderate and vigorous intensity activity, d) convey that physical activity does not have to be traditional gym activities (i.e. running on a treadmill), and e) clarify how to build in activities they enjoy.
**Motivational Messages**	Motivational messages should a) focus on how good you can feel as a result of following the guidelines, b) emphasize the enjoyment aspect of physical activity such as how to incorporate physical activity into activities that they enjoy such as spending time with friends, c) address self-regulation, and d) close with a call to action emphasizing that adults can get physical activity in many ways.
	*Examples*
	Feel better today!
	Catch up with a friend, take a walk!
	Set your goal, enjoy physical activity your way!
**Channels of Dissemination**	Messages should be disseminated through formerly inactive peers and through downloadable electronic tools available at partner websites that assist with self-regulation. Avoid the use of cartoons.

Key References

Anderson, E. S., Wojcik, J. R., Winett, R. A., & Williams, D. M. (2006). Social-cognitive determinants of physical activity: The influence of social support, self-efficacy, outcome expectations, and self-regulation among participants in a church-based health promotion study. *Health Psychology, 25* (4), 510-520.

Berry, T. R., Witcher, C., Holt, N. L., & Plotnikoff., R. C. (2010). A qualitative examination of perceptions of physical activity guidelines and preferences for format. *Health Promotion Practice. 11* (6), 908-916.

French, D. P., Sutton, S., Hennings, S. J., Mitchell, J., Wareham, N.J., Griffin, S.,…Kinmouth, A. L. (2005). The importance of affective beliefs and attitudes in the Theory of Planned Behavior: Predicting intention to increase physical activity. *Journal of Applied Social Psychology, 35* (9), 1824–1848.

Rhodes, R. E., Fiala, B., & Conner, M. (2009). A review and meta-analysis of affective judgments and physical activity in adult populations. *Annals of Behavioral Medicine, 38*(3), 180-204.

Rhodes, R. E. & Pfaeffi, L. A. (2010). Mediators of physical activity behavior change among adult non-clinical populations: A review update. *International Journal of Behavioral Nutrition and Physical Activity. 7*(1), 37.

Schwarzer, R. (2008). Modeling health behavior change: How to predict and modify the adoption and maintenance of health behaviors. *Applied Psychology, 57*(1), 1-29.

**Table 6:** Messaging recommendations for the new Canadian Physical activity Guidelines for Older Adults

	
**Preamble**	
These recommendations are relevant to all practitioners and organizations communicating the new Canadian Physical Activity Guidelines for Older Adults. These recommendations should be used to create a clear understanding of the new Canadian Physical Activity Guidelines for Older Adults and to construct and to deliver messages that promote achievement of the guidelines.	
**Recommendations**	
**Target Audience**	Messages should target apparently healthy older adults aged 65 years and over, health professionals and familial caregivers
**Clarification Messages**	Clarification messages should a) clarify that the recommended levels of physical activity can be done in self-selected blocks, b) provide “how to” information for key pieces of the guidelines that is presented in language that the older adult can understand, c) provide clear and concise descriptions of levels of intensity of physical activity, d) distinguish between walking and walking for exercise, e) provide examples of types of muscle and strength training exercises, and f) clarify the intensity level required for muscle and strength training exercises.
**Motivational Messages**	Motivational messages should a) focus on motivating older adults to engage in physical activity of moderate intensity or higher, b) aim to make older adults aware that vigorous activity is something of which they are capable, c) convey that physical activity can be fun and enjoyable, d) target confidence and concerns about becoming active later in life, e) highlight perceived barriers that may not be real barriers, such as age and experience, and f) highlight benefits of physical activity that are important to older adults.
	*Examples*
	Seniors can sweat.
	Heavy breathing is not just for the young.
	Enjoy an active life! You’ve earned it!
	It may be more fun than you think.
	The world can be your gym
	Not/never too late to start! No experience required!
	Stay fit. Stay independent. Stay connected
**Channels of Dissemination**	Messages should be disseminated through a) physicians and other health professionals (e.g., nurses, PTs, OTs, recreation therapists in nursing homes) via the web (CSEP, ParticipACTION, specialty organizations for the each of the health professions), b) the local government (i.e., departments within municipalities that deal with healthy living/seniors) via the web, public relations and other traditional outreach programs/activities, c) printed brochures distributed to physicians, health professionals, activity coordinators, or directly to older adults (e.g., community level organizations for seniors, lions clubs, seniors days, physical activity events), and d) the Canadian Association of Gerontology.

Key References

Ashford, S., Edmunds, J. & French, D.P. (2010). What is the best way to change self-efficacy to promote lifestyle and recreational physical activity? A systematic review with meta-analysis. *British Journal of Health Psychology*, 15, 265–288. doi: http://10.1348/135910709X461752.

Brawley, L. & Latimer, A. E. (2007). Physical activity guides for Canadians: messaging strategies, realistic expectations for change and evaluation. *Applied Physiology, Nutrition, and Metabolism*, 32, S170-S184

Dechaine, J & Witcher, C. (2007). Rural Route to Active Aging Focus Group Report: What We Heard in Rural Alberta. Retrieved from http://www.centre4activeliving.ca/older-adults/rural/focus-report.pdf

Hardy, S. & Grogan, S. (2009). Preventing disability through exercise: Investigating older adults’ influences and motivations to engage in physical activity. *Journal of Health Psychology October*, 14, 1036-1046. doi: http://10.1139/H07-105

Jones, L.W., Sinclair, R.C., and Courneya, K.S. (2003). The effects of source credibility and message framing on exercise intentions, behaviors, and attitudes: An integration of the elaboration likelihood model and prospect theory. *Journal of Applied Social Psychology*, 33, 179–196. doi: http://10.1111/j.1559-1816.2003.tb02078.x.

Paterson, D. H., & Warburton, D. E. (2010). Physical activity and functional limitations in older adults: A systematic review related to Canada's Physical Activity Guidelines. *International Journal of Behavioral Nutrition and Physical Activity, 7*, 38. doi: http://10.1186/1479-5868-7-38.

Peters, W. (2010). Age-related changes in decision making. In A. Drolet, N. Schwarz, & Yoon, C. (Eds.), The aging consumer. *Perspectives from psychology and economics* (pp. 75-101).

Rhodes, R., Fiala, B., & Connor, M. (2009). A review and meta-analysis of affective judgments and physical activity in adult populations. *Annals of Behavioral Medicine*, 38(3), 180-204. doi http://10.1007/s12160-009-9147-y

Berry, T. Spence JC, Plotnikoff, R.C.… Stolp, S. (2009). A mixed methods evaluation of televised health promotion advertisements targeted at older adults. *Evaluation and Program Planning*, 32, 278-288. doi.org/http://10.1016/j.evalprogplan.2009.05.001.

### AGREE domain 2: stakeholder involvement

The CSEP, ParticipACTION, and the PHAC in consultation with the lead researchers determined the key stakeholders (end users) of the recommendations. The recommendations specifically were directed towards organizations (government and non-governmental) and individual health professionals using the CPAG to promote physical activity participation.

Key stakeholders were involved at multiple points throughout the process. For example, the project was directed by CSEP, ParticipACTION, and PHAC – with input from researchers with expertise in theoretical approaches to physical activity promotion and messaging. Twenty-eight experts from across Canada participated in small workgroups during a 2-day meeting. Experts included researchers from multiple disciplines, health professionals, and representatives from government and non-governmental organizations. The table included in Additional file 8 lists workgroup members, their expertise, and their roles in the guideline development process. Once developed, the recommendations were reviewed through an online consultation by 81 end users.

### AGREE domain 3: rigor of development

#### The evidence-base

The evidence-base included systematic reviews related to physical activity determinants, message development, and message delivery [14-16], articles specific to messaging in Canada, and examples of successful comprehensive messaging campaigns [17-22]. The evidence-base was developed through purposeful article selection by the project directors rather than systematic review methods. This approach was used because multiple, up-to-date systematic reviews applicable to message development already were available and some context-specific data had recently been collected. Of benefit, this approach allowed expeditious preparation of the evidence-base and the inclusion of evidence specific to the Canadian context; yet this approach was susceptible to selection bias. The evidence selection methods were as follows:

a) The systematic reviews prepared in Phase 1 of the project served as the focal evidence for the recommendations. In a further attempt to ensure broad inclusion of relevant literature and to minimize bias, the evidence-base also included additional review articles that were identified by the project directors while preparing their systematic reviews in Phase 1.

b) A sample of articles reporting the success of past efforts to communicate the guidelines in Canada and of physical activity mass media campaigns in North America were selected from personal libraries. To reduce potential selection bias, the expert workgroup members were asked to identify any additional published messaging research specific to the North American context. No additional articles were circulated.

c) The evidence base also included a summary of findings from a series of public consultations and a nation-wide survey regarding stakeholders’ preferences for the delivery of messages supplementing the new CPAG. The consultations and survey were completed by the PHAC just prior to the meeting. A representative from the PHAC delivered a presentation at the start of the meeting summarizing key findings related to Canadians’ preferred messengers and format for delivering the new CPAG *and* supplemental messages.

The strengths and limitations of the messaging evidence were considered extensively in each of the articles that the workgroups were provided. Common strengths of the evidence were: a) the application of a theoretical framework to guide physical activity behavior change (e.g.,[16,17]); b) the availability of Canadian data describing previous efforts to disseminate physical activity guidelines (e.g.,[18,19] ); and c) the use of large population-based samples to monitor message uptake (e.g., [20,21] ). Common limitations included: a) the reliance on self-report measures to assess primary outcome variables (e.g., [15,22]); b) the lack of evidence supporting theoretical mediators of behavior change resulting from interventions with low fidelity and no initial pilot testing (e.g.,[15-17]); and c) the restricted generalizability of the study findings to the larger population (many of the intervention studies used to inform our recommendations included samples of middle-aged women) [15]. As such, the quality of the messaging evidence was not assessed systematically. Rather, we relied on the expert panel to consider the strengths and limitations of the evidence as identified within each article and to judge the appropriateness of the evidence for informing the messaging recommendations.

In cases where the evidence provided did not adequately support a messaging recommendation, workgroup members were asked to seek out supplemental evidence from the peer-reviewed scientific literature. The quality of this supplemental evidence was not assessed directly; instead, expert panel members determined its appropriateness for use. For example, the work group focusing on the CPAG for children used several supplemental references to support the important role of parents in promoting physical activity in this age group.

### Recommendation development

We developed the messaging recommendations through a multi-step process. One week in advance of the meeting, delegates were provided with a set of four to six articles from the evidence base to review. The resources provided to each workgroup are listed in a table included in Additional file 2. At the start of the meeting the project directors also delivered a 30-minute presentation summarizing key points to consider from the evidence base and the PHAC representative informed the group of the findings from their recent consultation process. Next, the workgroups participated in a series of structured discussions to formulate the guidelines. The workgroups were required to support each of their discussion points using the evidence base provided or supplemental evidence they had sought out independently. The larger group reviewed the key discussion points that emerged from each workgroup. Following the meeting, a summary document was circulated to the workgroups for review and revision. All recommendations and their associated evidence included in the final document are described in Additional files 3, 4, 5, 6, 7.

### Implications and updates

The recommendations have direct practical implications. The practical benefit is that they are the first set of evidence-based recommendations for creating messages supplementing physical activity guidelines. The application of these recommendations to practice has the potential to increase the effectiveness of the uptake of physical activity guidelines through the creation of message-based resources and tools.

The larger group also discussed procedures for updating the messaging recommendations. Due to resource limitations within the end users’ organizations, regular updates to the messages in the absence of a subsequent revision to the CPAG were not viewed as feasible. The consensus was that there already were limited resources for the release of the new CPAG let alone for constantly updating materials promoting the guidelines. The CPAG are slated for review and update every three to five years [10]. This would be optimal timing for updating the messaging recommendations as well; likely new clarification messages would be necessary at minimum.

### AGREE domain 4: clarity of presentation

For each CPAG, multiple recommendations were provided to allow flexibility in formulating messages. To create a concise resource for practitioners, the first author extracted these recommendations from the final summary document and created a series of information sheets. The information sheets include the recommendations and a preamble stating the objective of the recommendations. These information sheets were evaluated through a web-based survey by 81 practitioners including representatives from all levels of government (36.9%), fitness professionals (20.0%), staff/board members from non-governmental organizations (18.5%), and allied health professionals (10.8%). Each recommendation was rated on a 7-point scale for clarity, comprehensiveness, and ease of use. Participants also indicated their agreement with the recommendation and their likelihood of using the recommendation in practice. Overall, the responses to the survey were positive with all means falling above the scale midpoint and ranging from 4.48 to 5.43.

There were several suggestions to identify additional message target groups and channels of dissemination. Many participants requested additional details, examples, and resources. The specific recommendations were not modified in accordance with these requests. There was not adequate evidence to extend beyond the target groups and channels of dissemination already identified in the recommendations. Providing additional examples and resources was beyond the parameters of the recommendations. To address this feedback, the scope of the recommendations was clarified in the general preamble added to the beginning of the recommendations.

### AGREE domain 5: applicability

#### Uptake

The applicability of the recommendations was thoroughly considered by the panel. Sample messages and possible channels for message dissemination were provided with the recommendations to facilitate their application to practice. The panel discussed potential barriers to implementing the messaging recommendations. Concerns were raised regarding how to transition between existing materials and new materials promoting the new CPAG. For example, some provincial organizations were in the middle of mass media campaigns that included messages and materials promoting the older guidelines. Uncertainty regarding the financial resources available to produce new materials to supplement the new CPAG and the lack of a comprehensive strategic plan for developing these materials were considered barriers to applying our messaging recommendations.

A subgroup of six experts was convened to discuss strategies for evaluating the dissemination of the new CPAG and supplemental materials. The use of existing data collection mechanisms (e.g., Canadian Fitness and Lifestyle Research Institute Physical Activity Monitor, ParticipACTION’s ongoing market survey) was recommended. The group also suggested using the hierarchy of effects model [23] and the RE-AIM framework [24] to guide the dissemination evaluation. Because no resources were allocated to either dissemination or evaluation, no specific plans were made to further develop an evaluation scheme.

### AGREE domain 6: editorial independence

The process was funded by CSEP and PHAC. Although these organizations participated in the process of developing the recommendations, their involvement did not influence the recommendations for creating messages. Their involvement did influence discussions related to facilitators and barriers to implementation, resource implications and evaluation. Delegates from these organizations were able to present the real challenges facing the release of the new CPAG and the production of supplemental materials. The depth of the discussion related to evaluation was blunted by uncertainties related to available funding.

None of the workgroup members declared a conflict of interest.

## Results

### Recommendations

Through a systematic process guided by the AGREE II, we developed evidence-based specific recommendations for constructing and disseminating messages supplementing the new CPAG for children, youth, adults, and older adults. Recommendations also were developed to raise awareness of the new guidelines in general guided process. The recommendations are summarized in Tables 1, 2, 3, 4, 5, 6. The rationale supporting these recommendations is included in the Additional files 3, 4, 5, 6, 7.

### Uptake

At the time of the consensus meeting, several opportunities for uptake of the messaging recommendations were anticipated through the three key stakeholder groups involved in the project. Initially, we intended our recommendations to inform the development of messages accompanying the CPAG when they were released in January 2011 (two months after the consensus meeting). This uptake did not come to fruition due to changes in our stakeholder involvement and delay in producing the final messaging recommendations.

While some supplementary materials were produced for the release of the CPAG, they were limited. Some of the materials did incorporate content that aligned with our recommendations.

## Discussion

### Strengths and limitations of our recommendations

To our knowledge, our efforts for constructing and disseminating messages supplementing the new CPAG represent the first set of evidence-informed recommendations for creating messages supplementing physical activity guidelines. Given that we drew from existing evidence of effective messaging interventions (e.g., VERB, ParticipACTION [22,25]), these recommendations represent an accumulation of best practices in the field of physical activity promotion, health communications, and public health intervention.

Although the messaging recommendations have potential for shaping practice, their limitations must be acknowledged. Our recommendations do not have direct health benefits– they target an intermediary group (message developers) rather than the end users per se. However, if practitioners effectively apply the recommendations, there are potential indirect effects on health because the resulting messages will promote physical activity in accordance with the new CPAG.

The recommendations are further limited as a result of methodological constraints – many of which could easily have been addressed by involving a guideline development methodologist as part of the research team from the onset. First, the evidence-base was determined by the primary authors with suggestions from expert panel members. It also included findings from an unpublished government report that included newly collected data critical to informing the guidelines. Rigorous systematic review methods that include evaluation of study quality and strict inclusion criteria are optimal.

Second, the messaging recommendations were not sent for external review outside of the panel of experts who participated in the development process. The panel had substantial breadth in expertise; nonetheless external reviewers might have suggested modifications to strengthen the recommendations. Had we opted for additional external review, we could have sought the opinions of international experts, health communication technology researchers, and marketing and advertising professionals as individuals with this type of expertise were underrepresented in our workgroups. Indeed, the addition of individuals with expertise in the application of technology for health communications would have brought forward emerging evidence of promising technology-based approaches for disseminating our messages. With regards to marketing experts, only one panel member had specific expertise in this area. Public health often neglects to consult with this highly knowledgeable group of professionals. This shortcoming is unfortunate as marketing experts have potential to enhance reach of messages within the target audience and assist in designing visual, graphic and print copy of the final messages.

Third, we did not formally engage a methodologist throughout our process to participate in evaluation of the evidence, and to assess our process [26]. However, herein we report our process as transparently as possible. The objective of AGREE II is to promote transparency in the guideline development process. To ensure transparency in this case study, all of the steps undertaken in developing the messaging recommendation in relation to the AGREE II are outlined in the table included in Additional file 1.

### The process of developing recommendations: successes, challenges and future directions

To our knowledge, this case study represents the first application of international standards for guideline development set out in the AGREE II Instrument to the development of practical recommendations specifically aimed to inform health promotion practice. Of benefit, the AGREE II Instrument provided a foundation for guiding the steps in the recommendation development process, for determining the make-up of our expert panel, and for reporting the process. The Instrument was quite easily adapted to suit our objective. Modifications primarily centered around shifting the guideline focus from patient groups to guideline message developers, using a modified evidence-review process, and considering the practical implications of the recommendations versus the direct health implications. With further development and validation, the adapted AGREE II Instrument has potential to become the standard for developing evidence-based health behavior change recommendations and, more generally, knowledge tools that facilitate the application of existing clinical practice guidelines.

We encountered practical challenges developing our messaging recommendations. Final messaging recommendations occurred over 1 year following our consensus meeting. The messaging consensus meeting was held in November 2010; the recommendations were not finalized until July 2011 and evaluated by stakeholders in January 2012 – more than one year following the consensus meeting. The lag could have been addressed by setting and adhering to clear time deadlines for the AGREE II steps following the consensus meeting.

Two factors impaired progress: a) the timeline for finalizing the recommendations and b) the absence of funds to develop messages supporting the guidelines. The timeline for developing recommendations was ambitious. The consensus panel meeting was held in November 2010, recommendations were needed by the key stakeholder groups almost immediately to begin production of messages to accompany the guidelines for a January 2011 launch. This timeline would have been feasible with adequate personnel to support the initiative; however, in the absence of resources, no personnel were available. As a temporary measure, the three key stakeholders were provided with a summary report from the expert panel to use in preparation for the guideline launch.

Finally, at the time of the consensus meeting it seemed promising that some resources would be allocated to developing materials supplementing the guidelines that would apply our recommendations. However, shortly after the meeting, the partnership between the three key stakeholders (CSEP, PHAC, ParticipACTION) dissolved. Plans for developing a comprehensive set of supplemental materials were halted and the immediate need for message construction recommendations was lessened.

As a result of these practical challenges, the application of the recommendations has been limited [27]. Much should be learned from this lack of uptake. While there was substantial interest and even excitement about producing recommendations to facilitate the application of CPAG, the lack of a feasible and sustainable plan for implementation hindered the application of the messaging recommendations. The dissolution of the partnership between the key stakeholders also dampened enthusiasm; the potential for implementation was substantially greater when all key stakeholders were committed to disseminating the CPAG and the accompanying motivational messages developed in accordance with our messaging recommendations in partnership. Our messaging recommendations represent advancement in knowledge translation efforts by researchers and stakeholders but highlight the fact that knowledge translation is a multi-step process that requires an enduring commitment of resources, financial and personnel, from all parties involved.

## Conclusions

There is growing demand for evidence-informed decision-making in the design and implementation of health behavior change programming. Practitioners are challenged to meet these demands in the absence of evidence and specifically of recommendations that consolidate available evidence into a usable knowledge tool. This case study documents a two-phased process for translating this research into practical recommendations. To our knowledge, these are the first set of evidence-informed recommendations for constructing and disseminating health behavior change messages supplementing clinical practice guidelines. This project also represents the first application of international standards, the AGREE II, for the development of recommendations specifically aimed to inform behavior change practice. Application of these standards to other health behavior change initiatives has potential to facilitate the process of translating research into practice, and consequently strengthening health promotion programming.

## Competing interests

The authors declare that they have no competing interests.

## Authors’ contributions

AELC, RER, MEK, and MD participated in the conception, design, and coordination of the study. ALC, RER, MEK and JRT helped to draft the manuscript. HLG assisted with manuscript revision. JRT, HLG, KK, GN, and MJP participated in the acquisition of data and study coordination. The CPAG Messaging Recommendation Workgroup developed and reviewed the recommendations. All authors read and approved the final manuscript.

## Pre-publication history

The pre-publication history for this paper can be accessed here:

http://www.biomedcentral.com/1471-2458/13/419/prepub

## Supplementary Material

Additional file 1: Table S1 Modifications to AGREE II items. The table lists the AGREE II items and includes descriptions of how the items were modified and applied in the case study. Click here for file

Additional file 2: Table S2 Resources provided to workgroups. This table lists the references for the materials provided to each workgroup. Click here for file

Additional file 3 **Supporting Evidence for the Messaging Recommendations for the New Canadian Physical Activity Guidelines for Overarching Messages.** This file provides the rationale and lists the supporting evidence for each of the overarching messaging recommendations. Click here for file

Additional file 4 **Supporting Evidence for the Messaging Recommendations for the New Canadian Physical Activity Guidelines for Children.** This file provides the rationale and lists the supporting evidence for the messaging recommendations for the CPAG for Children. Click here for file

Additional file 5 **Supporting Evidence for the Messaging Recommendations for the New Canadian Physical Activity Guidelines for Youth.** This file provides the rationale and lists the supporting evidence for the messaging recommendations for the CPAG for Children. Click here for file

Additional file 6 **Supporting Evidence for the Messaging Recommendations for the New Canadian Physical Activity Guidelines for Adults.** This file provides the rationale and lists the supporting evidence for the messaging recommendations for the CPAG for Children. Click here for file

Additional file 7 **Supporting Evidence for the Messaging Recommendations for the New Canadian Physical Activity Guidelines for Older Adults.** This file provides the rationale and lists the supporting evidence for the messaging recommendations for the CPAG for Older Adults. Click here for file

Additional file 8: Table S3 Expert panel. This table lists the names, expertise, and roles of the individuals who participated in the recommendation development meeting. Click here for file
