# Supplementary material for: Evidence-informed recommendations for constructing and disseminating messages supplementing the new Canadian Physical Activity Guidelines
Source: BMC Public Health. 2013 May 1;13:419. doi: 10.1186/1471-2458-13-419 (PMC3654879; doi:10.1186/1471-2458-13-419)
Supplement: Additional file 1: Table S1 — Modifications to AGREE II items. The table lists the AGREE II items and includes descriptions of how the items were modified and applied in the case study. [file 1471-2458-13-419-S1.docx]

# Table 7. Modifications to AGREE II items

| AGREE II item | Modified AGREE II item | Application to the current project |
| --- | --- | --- |
| ***Domain 1: Scope and Purpose*** | | |
| 1. The overall objective(s) of the guideline is (are) specifically described. | The overall objective(s) of the message recommendations is (are) specifically described. | To develop recommendations for the optimal content, target audience, and channels of dissemination for messages supplementing the new CPAG. |
| 1. The health question(s) covered by the guideline is (are) specifically described. | The practical question(s) covered by the message recommendations is (are) specifically described. | 1) What information should be conveyed to Canadians to raise their awareness of the new guidelines?, 2) What aspects of the guidelines need clarification?, 3) What is the optimal content for motivational messages?, 4) Who are the key people to receive these messages?, and 5) How should these messages be disseminated? |
| 1. The population (patients, public, etc.) to whom the guideline is meant to apply is specifically described. | The population to whom the message recommendations are targeted is specifically described. | Target populations for each of the messages are specific to each CPAG (child, youth, adult, older adult). |
| ***Domain 2: Stakeholder Involvement*** | | |
| 1. The guideline development group includes individuals from all relevant professional groups. | The message recommendations development group includes individuals from all relevant professional groups. | Each workgroup was made up of experts in communications, exercise physiology, physical activity behavior change and messaging, and representatives from federal and provincial government healthy living agencies. |
| 1. The views and preferences of the target population (patients, public, etc.) have been sought. | The views and preferences of the target population (i.e., end users) have been sought. | Workgroups included end users of messaging recommendations. The recommendations were reviewed by 81 end users. |
| 1. The target users of the guideline are clearly defined. | The target users of the messages are clearly defined. | The target populations for the message recommendations are organizations (government and non-governmental) and individual health professionals using the CPAG to promote physical activity participation. |
| ***Domain 3: Rigour of Development*** | | |
| 1. Systematic methods were used to search for evidence. | Systematic methods were used to search for evidence. | Articles were purposely selected by the project directors including the systematic reviews from Phase 1 and multiple, up-to-date systematic reviews applicable to message development were already available and some context-specific data had recently been collected. |
| 1. The criteria for selecting the evidence are clearly described. | The criteria for selecting evidence are clearly described. | Recent systematic reviews related to physical activity determinants, message development, and message delivery, as well as all articles reporting the success of past efforts to communicate the guidelines in Canada and of physical activity mass media campaigns in Canada and the United States were selected. |
| 1. The strengths and limitations of the body of evidence are clearly described. | The strengths and limitation of the body of evidence are clearly described. | Common strengths of the evidence were: a) the application of a theoretical framework to guide physical activity behavior change, b) the availability of Canadian data describing previous efforts to disseminate physical activity guidelines, and c) the use of large population-based samples to monitor message uptake. Common limitations were: a) the reliance on self-report measures to assess primary outcome variables, b) the lack of evidence supporting theoretical mediators of behavior change resulting from interventions with low fidelity and no initial pilot testing, and c) the restricted generalizability of the study findings to the larger population. |
| 1. The methods for formulating the recommendations are clearly described. | The methods for formulating the recommendations are clearly described. | Multi-step process: 1) Evidence reviewed in advance of two-day meeting, 2) 30-minute presentations summarizing key points from evidence base, 3) structured discussions in workgroups, 4) review of key discussion points with group as a whole, 5) provision of summary document of messaging recommendations and description of evidence-based rationale, 6) review and revision of recommendations by workgroup members. |
| 1. The health benefits, side effects, and risks have been considered in formulating the recommendations. | The direct practical implications have been considered in formulating the recommendations. | Indirect effects on health are possible because the recommendations themselves will be applied to develop messages promoting physical activity in accordance with the new CPAG. Practical implications of these recommendations include a potential to increase the effectiveness of the materials being created. |
| 1. There is an explicit link between the recommendations and the supporting evidence. | There is an explicit link between the messaging recommendations and the supporting evidence. | The rationale and evidence-base for each recommendation is clearly outlined in Additional Files 4-8. |
| 1. The guideline has been externally reviewed by experts prior to its publication. | The message recommendations have been externally reviewed by experts prior to its publication. | Message recommendations were reviewed and revised by workgroup experts and pilot tested among end users. |
| 1. A procedure for updating the guideline is provided. | A procedure for updating the message recommendations is provided. | Message recommendations (especially those for clarification messages) should be updated at the same time as guideline updates (i.e., every 5 years). However, this may not be financially feasible. |
| ***Domain 4: Clarity of Presentation*** | | |
| 1. The recommendations are specific and unambiguous. | The recommendations are specific and unambiguous. | The recommendations were considered clear in a pilot test by end users |
| 1. The different options for management of the condition or health issue are clearly presented. | The different options for developing and disseminating messages are clearly presented. | Multiple recommendations are provided to allow for flexibility in formulating messages. |
| 1. Key recommendations are easily identifiable. | Key recommendations are easily identifiable. | Information sheets clearly outline key messaging recommendations for each CPAG target group. |
| ***Domain 5: Applicability*** | | |
| 1. The guideline describes facilitators and barriers to its application. | Facilitators and barriers to the application of the recommendations were discussed. | The group as a whole discussed barriers to implementation. Additional barriers and facilitators are provided in the case study conclusion. |
| 1. The guideline provides advice and/or tools on how the recommendations can be put into practice. | The message recommendations provide advice and/or tools on how the recommendations can be put into practice. | Sample messages and possible channels for message dissemination are provided to facilitate the application of the recommendations to practice. |
| 1. The potential resource implications of applying the recommendations have been considered. | The potential resource implications of applying the recommendations have been considered. | Group as a whole discussed the financial implications (i.e., salary and production costs) required for developing new materials that applied the recommendations. |
| 1. The guideline presents monitoring and/or auditing criteria. | Strategies for monitoring and/or auditing the uptake of the recommendations was considered by the expert panel. | Evaluation and monitoring strategies were discussed but are not included in message recommendations. Lack of financial resources available for evaluation limited the necessity of this discussion. |
| ***Domain 6: Editorial Independence*** | | |
| 1. The views of the funding body have not influenced the content of the guideline. | The views of the funding body have not influenced the content of the message recommendations. | Representatives from funding agencies (CSEP and PHAC) participated but did not influence message recommendations. |
| 1. Competing interests of guideline development group members have been recorded and addressed. | Competing interests of message recommendation development group members have been recorded and addressed. | None of the workgroup members reported conflicts of interest. |
