# Supplementary material for: Evidence-informed recommendations for constructing and disseminating messages supplementing the new Canadian Physical Activity Guidelines
Source: BMC Public Health. 2013 May 1;13:419. doi: 10.1186/1471-2458-13-419 (PMC3654879; doi:10.1186/1471-2458-13-419)
Supplement: Additional file 2: Table S2 — Resources provided to workgroups. This table lists the references for the materials provided to each workgroup. [file 1471-2458-13-419-S2.docx]

# Table 9. Resources provided to workgroups

| ***Children and Youth Resources*** |
| --- |
| 1. Craig CL, Bauman A, Gauvin L, Robertson J, Murumets K: **ParticipACTION: A mass media campaign targeting parents of inactive children; knowledge, saliency, and trialing behaviours.** *Int J Behav Nutr Phy* 2009, **6:**88. 2. Lubans DR, Foster C, Biddle SJ: **A review of mediators of behavior in interventions to promote physical activity among children and adolescents.** *Preventive Medicine* 2008, **47:**463-470. 3. van Sluijs EM, McMinn AM, Griffin SJ: **Effectiveness of interventions to promote physical activity in children and adolescents: systematic review of controlled trials.** *BMJ* 2007, **335:**703-715. 4. Wong F, Huhman M, Asbury L, Bretthauer-Mueller R, McCarthy S, Londe P, Heitzler C: **VERB™—a social marketing campaign to increase physical activity among youth**. *Prev Chronic Dis* 2004, **1**. |
| ***Adult and Older Adult Resources*** |
| 1. Brawley LR, Latimer AE: **Physical activity guides for Canadians: messaging strategies, realistic expectations for change, and evaluation.** *Appl Physiol Nutr Me* 2007, **32:**S170-S184. |
| 1. Latimer AE, Brawley LR, Bassett RL: **A systematic review of three approaches for constructing physical activity messages: What messages work and what improvements are needed?** *Int J Behav Nutr Phys Act* 2010, **7:**36. |
| 1. Rhodes RE, Pfaeffli LA: **Mediators of physical activity behaviour change among adult non-clinical populations: a review update.** *Int J Behav Nutr Phy* 2010, **7:**37. |
|  |
| ***Evaluation Resources*** |
| 1. Cameron C, Craig C, Bull F, Bauman A: **Canada’s physical activity guides: Has their release had an impact?** *Appl Physiol, Nutr, and Metab* 2007, **32:**161-169. |
| 1. Spence JC, Brawley LR, Craig CL, Plotnikoff RC, Tremblay MS, Bauman A, Faulkner GEJ, Chad K, Clark MI: **ParticipACTION: Awareness of the participACTION campaign among Canadian adults - Examining the knowledge gap hypothesis and a hierarchy-of-effects model.** *Int J Behav Nutr Phy* 2009, **6:**85. |
| ***Dissemination Resources*** |
| 1. Berry TR, Witcher C, Holt NL, Plotnikoff RC: **A Qualitative Examination of Perceptions of Physical Activity Guidelines and Preferences for Format.** *Health PromotPract* 2008. **11:** 906-916. |
| 1. Marshall A, Owen N, Bauman A: **Mediated approaches for influencing physical activity: Update of the evidence on mass media, print, telephone and website delivery of interventions.** *J Sci Med Sport* 2004*,* **7:**74-80. |
| 1. van den Berg MH, Schoones JW, Vliet Vlieland TP: **Internet-based physical activity interventions: a systematic review of the literature.** *J Med Internet Res* 2007, **9:**e26. |
