# Supplementary material for: Evidence-informed recommendations for constructing and disseminating messages supplementing the new Canadian Physical Activity Guidelines
Source: BMC Public Health. 2013 May 1;13:419. doi: 10.1186/1471-2458-13-419 (PMC3654879; doi:10.1186/1471-2458-13-419)
Supplement: Additional file 3 — Supporting Evidence for the Messaging Recommendations for the New Canadian Physical Activity Guidelines for Overarching Messages. This file provides the rationale and lists the supporting evidence for each of the overarching messaging recommendations. [file 1471-2458-13-419-S3.docx]

# Supporting Evidence for the Messaging Recommendations for the New Canadian Physical Activity Guidelines for Overarching Messages

*In text citation numbers correspond with the reference list at the end of Additional File 4

## Target Audience

| Messages should target… | Rationale |
| --- | --- |
| All Canadians | Everyone needs to be made aware that new physical activity guidelines are being released. |

## Awareness Messages

| Overarching awareness messages should… | Rationale | Example(s) |
| --- | --- | --- |
| Inform Canadians that there are new guidelines based on scientific evidence | Canadians need to know that there are new PA guidelines and that they are different from the guidelines released in 1998 as a result of recent research evidence. | Canada has new PA guidelines to help you understand how to live a healthier life.  Canada has new PA guidelines to help you understand how to live a healthier life. |
| Convey that rigorous, scientific reviews have revealed new information about the health benefits of physical activity | We did not want Canadians to feel as though the old guidelines were obsolete or false. We wanted to stress that a diligent/rigorous research process was followed so to include the most up-to-date and evidence-based information in the new guidelines. |  |

## Clarification Messages

| **Clarification messages should…** | **Rationale** | **Example(s)** |
| --- | --- | --- |
| Explain why the guidelines have changed | Systematic reviews [1-5] examined hundreds of papers re: guidelines and messaging strategies have revealed new information about physical activity recommendations for health benefits.  Need separate messages for two target audiences: lay public and health professional/ policy maker. Whereas health professionals and policy makers would want to know more information about the rigorous, evidence-based process that was used, the lay public just needs to understand that the guidelines have changed because of new scientific evidence.  Want health professionals and policy makers to know that we followed AGREE II process for clinical guideline development. | Lay public message 🡪 Canada’s new physical activity guidelines reflect the most up-to-date research on the health benefits of physical activity.  Health professional/policy makers 🡪 Canada’s new physical activity guidelines are the result of a transparent/rigorous/systematic guideline development process that ensures the guidelines are unbiased and credible. This is the first physical activity guideline development process in the world to be guided by the AGREE II Instrument. |
| Clarify terminology included in the guidelines | Some terminology contained in the 1998 guidelines was ambiguous.  Want to ensure that all Canadians were using consistent definitions so that the new guidelines were not left open to interpretation. | Please refer to the attached document “Canadian Physical Activity Guidelines: Glossary of Terms” for definitions and examples of the following terms*:   - - Physical activity   - Physical activity guidelines   - Aerobic physical activity   - Apparently healthy (separate definitions for children and youth, adults, and older adults)   - Balance enhancement or balance training   - Bone-strengthening activity   - Bout   - Duration   - Frequency   - Health professional   - Incidental activity   - Intensity (as well as absolute intensity and relative intensity)   - Moderate-intensity physical activity   - Vigorous-intensity physical activity   - Muscle-strengthening activity |

## References

1. Janssen I, Leblanc AG: **Systematic review of the health benefits of physical activity and fitness in school-aged children and youth.** *Int J Behav Nutr Phys Act* 2010, **7:**40.
2. Latimer AE, Brawley LR, Bassett RL: **A systematic review of three approaches for constructing physical activity messages: What messages work and what improvements are needed?** *Int J Behav Nutr Phys Act* 2010, **7:**36.
3. Paterson DH, Warburton DE: **Physical activity and functional limitations in older adults: a systematic review related to Canada's Physical Activity Guidelines.** *Int J Behav Nutr Phys Act* 2010, **7:**38.
4. Rhodes RE, Pfaeffli LA: **Mediators of physical activity behaviour change among adult non-clinical populations: a review update.** *Int J Behav Nutr Phy* 2010, **7:**37.
5. Warburton DE, Charlesworth S, Ivey A, Nettlefold L, Bredin SS: **A systematic review of the evidence for Canada's Physical Activity Guidelines for Adults.** *Int J Behav Nutr Phys Act* 2010, **7:**39.

*Definitions for glossary terms were taken from either:

Centers for Disease Control and Prevention: Physical activity for everyone: Glossary of terms. [http://www.cdc.gov/physicalactivity/everyone/glossary/index.html]

World Health Organization: Global recommendations on physical activity for health. [http://www.who.int/dietphysicalactivity/factsheet_recommendations/en/index.html]
