# Supplementary material for: Evidence-informed recommendations for constructing and disseminating messages supplementing the new Canadian Physical Activity Guidelines
Source: BMC Public Health. 2013 May 1;13:419. doi: 10.1186/1471-2458-13-419 (PMC3654879; doi:10.1186/1471-2458-13-419)
Supplement: Additional file 4 — Supporting Evidence for the Messaging Recommendations for the New Canadian Physical Activity Guidelines for Children. This file provides the rationale and lists the supporting evidence for the messaging recommendations for the CPAG for Children. [file 1471-2458-13-419-S4.docx]

# Supporting Evidence for the Messaging Recommendations for the New Canadian Physical Activity Guidelines for Children

*In text citation numbers correspond with the reference list at the end of Additional File 5

## Target Audience

| Messages should target… | Rationale |
| --- | --- |
| Teachers | Children spend most of their day at school; therefore it is imperative for teachers to act as role models throughout the school day by demonstrating physical activity and fostering positive attitudes toward physical activity [1,2]. Teachers can also help meet the unsatisfied need of increasing the amount of time that children are physically active during physical education [3]. Teachers can control the curriculum to encourage and embed physical activity throughout the day. Teachers can also advocate for comprehensive school health policies outside of normal classroom activities creating an “activity culture” at the school. |
| Parents | Parents have the opportunity, through socialization, to shape their child’s interests and attitude [4], as well as to encourage and role model whole family physical activity [3]. Furthermore, it has been shown that parental enjoyment of physical activity, parental physical activity and parental support are positively associated with child physical activity [5,6]. Parents can support physical activity through purchasing toys that promote activity and by facilitating registration in and transport to physical activity and sport venues [3]. |
| Children | Children, when prompted and encouraged to be active tend to be more active. This mirrors the marketing and advertising literature which highlights the responsive effects amongst children in response to targeted media and advertising campaigns (e.g., [7]). In the context of physical activity, trials such as the VERB (mass-media) campaign were found to result in higher physical activity and positive attitudes towards physical activity. Although this was a multi-modal intervention (also involving the community and school), as Huhman et al. [8] highlight, “television advertising, placed mainly on cable networks popular with children, was the primary delivery vehicle for the intervention”. Include some of the peer-literature in the 2011 Active Healthy Kids Canada Report Card [3]. |

## Clarification Messages

Clarification messages should…

- Help parents and teachers understand exactly what the guidelines are and that their role is pivotal in the physical activity that children engage in.
- Enable teachers and parents to help children understand, learn, and potentially make a habit of being active for a minimum of 60 minutes every day, and incorporating muscle and bone strengthening activities at least three times in a week.

## Motivational Messages

| Motivational messages should… | Rationale | Example(s) |
| --- | --- | --- |
| Encourage teachers to act as role models, promoting physical activity throughout the school day. | [1] Incorporating physical activity throughout the school day can help children focus and concentrate, as well as increase the feeling of school connectedness among children [9]. |  |
| Reinforce the parent’s pivotal role in shaping their child’s interests and attitudes. The messages should also reinforce the importance of planning to be physically active with the family. | Parents have a pivotal role in shaping their child’s interests and attitudes [4] and their support is positively associated with their child’s physical activity [5].  Plan to be active with the family [10]. | Invest in your child’s future by planning for physical activity now |
| Be fun, cool, and socially appealing to children. These messages should also target self-efficacy. | [11]  Targeting self-efficacy is a useful strategy when promoting physical activity behavior [12]. | Get active! It’s fun!  You CAN do it, it’s easy! |

## Channels of Delivery

| Messages should be disseminated through… | Rationale |
| --- | --- |
| Mass media (parents) | The ParticipACTION campaign effectively reached parents of elementary school-aged children, and as a result of having viewed the ParticipACTION commercials, more than a quarter of the parents had started to incorporate more physical activity into their and/or their family’s routine, 33% had talked to their child about being more physically active, and 20% had made stricter rules about the amount of time their child spent doing sedentary activities [13].  Parental awareness of the VERB campaign was predictive of positive attitudes about physical activity for children in general, belief in the importance of physical activity for their own child, and the number of days that they were active with their child [14]. |
| Worksheets (Teachers) | Provide practical resources on how to increase physical activity throughout the school day (e.g [15]). Can also provide tools to give to students. |
| Nongovernmental organizations (e.g., Boys and Girls’ Clubs; children) | Youth advocacy groups in the communities and in schools (e.g., with respect to substance use [16]). |
| Media and social media | [8]; social media |

| References  1. Dobbins M, De Corby K, Robeson P, Husson H, Tirilis D: **School-based physical activity programs for promoting physical activity and fitness in children and adolescents aged 6-18.** *Cochrane Database Syst Rev* 2009**:**CD007651. 2. Trudeau F, Shephard RJ: **Contribution of school programmes to physical activity levels and attitudes in children and adults.** *Sports Med* 2005, **35:**89-105. 3. Active Healthy Kids Canada: 2011 Report Card. [http://www.activehealthykids.ca/ReportCard/School.aspx] 4. Welk GJ, Wood K, Morss G: **Parental influences on physical activity in children: An exploration of potential mechanisms.** *Pediatr Exerc Sci* 2003, **15:**19-33. 5. Van Der Horst K, Paw MJ, Twisk JW, Van Mechelen W: **A brief review on correlates of physical activity and sedentariness in youth.** *Med Sci Sports Exerc* 2007, **39:**1241-1250. 6. Zecevic CA, Tremblay L, Lovsin T, Michel L: **Parental Influence on Young Children's Physical Activity.** *Int J Pediatr* 2010, **2010:**46852 7. Oates C, Blades M, Gunter B: **Marketing to children**. *Journal of Marketing Management* 2003, **19**:401-409. 8. Huhman ME, Potter LD, Duke JC, Judkins DR, Heitzler CD, Wong FL: **Evaluation of a national physical activity intervention for children: VERB campaign, 2002-2004.** *American Journal of Preventive Medicine* 2007, **32:**38-43 9. Active Healthy Kids Canada: 2009 Report Card. [http://www.activehealthykids.ca/ecms.ashx/ReportCard2009/AHKC-Longform_WEB_FINAL.pdf] 10. Rhodes RE, Naylor PJ, Mckay HA: **Pilot study of a family physical activity planning intervention among parents and their children.** *J Behav Med* 2010, **33:**91-100. 11. Huhman M, Potter LD, Wong FL, Banspach SW, Duke JC, Heitzler CD: **Effects of a mass media campaign to increase physical activity among children: year-1 results of the VERB campaign.** *Pediatrics* 2005, **116:**e277-e284. 12. Lubans DR, Foster C, Biddle SJ: **A review of mediators of behavior in interventions to promote physical activity among children and adolescents.** *Preventive Medicine* 2008, **47:**463-470. 13. Craig CL, Bauman A, Gauvin L, Robertson J, Murumets K: **ParticipACTION: A mass media campaign targeting parents of inactive children; knowledge, saliency, and trialing behaviours.** *Int J Behav Nutr Phy* 2009, **6**:88. |
| --- |
| 1. Price SM, Huhman M, Potter LD: **Influencing the parents of children aged 9-13 years: findings from the VERB campaign.** *American Journal of Preventive Medicine* 2008, **34:**S267-274. |
| 1. Naylor PJ, Macdonald HM, Zebedee JA, Reed KE, Mckay HA: **Lessons learned from Action Schools! BC - An 'active school' model to promote physical activity in elementary schools.** *J Sci Med Sport* 2006, **9:**413-423. 2. Tencati E, Kole SL, Feighery E, Winkleby M, Altman DG: **Teens as advocates for substance use prevention: Strategies for implementation**. *Health Promotion Practice* 2002*,* **3:**18-29. |
|  |
