# Supplementary material for: Evidence-informed recommendations for constructing and disseminating messages supplementing the new Canadian Physical Activity Guidelines
Source: BMC Public Health. 2013 May 1;13:419. doi: 10.1186/1471-2458-13-419 (PMC3654879; doi:10.1186/1471-2458-13-419)
Supplement: Additional file 5 — Supporting Evidence for the Messaging Recommendations for the New Canadian Physical Activity Guidelines for Youth. This file provides the rationale and lists the supporting evidence for the messaging recommendations for the CPAG for Children. [file 1471-2458-13-419-S5.docx]

# Supporting Evidence for the Messaging Recommendations for the New Canadian Physical Activity Guidelines for Youth

*In text citation numbers correspond with the reference list at the end of Additional File 6

## Target Audience

Targeting both parents and youth is integral for promoting the physical activity guidelines for youth. The physical activity guidelines for youth are targeted to a wide age range (ages 12-17). Given the various levels of maturation and parental involvement within this age range, we recommend two different campaigns for younger and older youth.

| Messages should target… | Rationale |
| --- | --- |
| Parents of youth aged 12-14. | Interventions that target the family of youth have been shown to affect youth physical activity levels [1,2]. |
| Youth aged 15-17. | Interventions that target youth directly have been shown to effective [2]. |
| Youth aged 12-14. | Interventions that target youth directly have been shown to effective [2]. |
| Parents of youth aged 15-17. | Interventions that target the family of youth have been shown to affect youth physical activity levels [1,2]. |

*Note.* Message targets are listed in order of importance.

## Clarification Messages

Clarification messages should…

- Provide concise and clear descriptions of the guidelines and physical activity.
- Define different levels of physical activity intensities (e.g., vigorous vs. moderate).
- Identify physical activity opportunities.
- Include information about where to access more information about physical activity.
- Indicate that science has evolved since the release of the old guidelines and that the new guidelines reflect latest evidence about physical activity.
- Address the semantic differences between the old and new guidelines (e.g., up to 90 minutes vs. at least 60 minutes).
- Provide a wide variety of examples of physical activity – from active living to sport.

## Motivational Messages

| Motivational messages should… | Rationale | Example(s) |
| --- | --- | --- |
| Underline the importance of parents as physical activity agents of change. | Parents have been identified in the literature as physical activity behavior agents for their children/youth [2-4]. | 60 minutes every day! As a parent, you can help your teen get there.  Move your teen to move more. |
| Focus on a variety of benefits that physical activity offers including emotional, developmental, physical and academic benefits. | Promoting the affective benefits of physical activity has been shown to affect physical activity behavior [5,6].  Evidence has shown that youth who engage in physical activity demonstrate stronger academic performance [7]. | Physically active kids get better grades.  Active teens are fit to learn. |
| Focus on the affective benefits of physical activity. | Promoting the affective benefits of physical activity has been shown to affect physical activity behavior [5,6]. | Life looks better with physical activity. |
| Emphasize that physical activity can be a collective-social movement that promotes community, active living and the environment. | ParticipACTION’s SOGO Active Campaign which focused on creating collective-social movements received positive feedback from youth.  Campaigns that have mobilized youth have shown greater dissemination of physical activity messages [2]. | Life looks better with physical activity. |
| Emphasize the importance of planning physical activity by parents and youth. | Lubans and colleagues [8] found that self-regulatory skills are an important predictor of physical activity. | Pick a time. Pick a place. Move your teens to move more.  Pick a time. Pick a place. How you want to get active is up to you! |
| Provide strategies for overcoming barriers to physical activity. | Rhodes & Pfaeffli [5] found that self-regulatory skills are an important predictor of physical activity. | Plan ahead for roadblocks! |
| Emphasize that physical activity can be an autonomous choice for teens. | Perceiving autonomy support from teachers, parents and peers over physical activity choices is related to leisure-time physical activity motivation [9]. | Expand your horizons! How you want to get active is up to you! |

## Channels of Delivery

| Messages should be disseminated through… | Rationale |
| --- | --- |
| Websites | Websites that target teens and parents have been shown increase the dissemination of physical activity messages and provide tools to help initiate and monitor physical activity [2]. |
| TV ads targeting parents. | Mass-media campaigns targeting parents of youth have been shown to effective [2,3] |
| Magazine and news articles. | Print-based messages have been shown to be an effective method for disseminating physical activity messages, can be more effective than web based messaging [10,11]. |
| Letters and posters distributed to teachers. | Print-based messages have been shown to be an effective method for disseminating physical activity messages [10]. |
| Celebrities | The use of celebrities within PSAs and televisions shows commonly watched by parents and teens have been shown to increase the marketing reach of physical activity campaigns [2]. |
| Social media | Given the widespread use of social media by Canadian [12], social media websites may be an effective way to communicate information to youth. |

| Smart phone applications | As many Canadians migrate to using smartphones [13], the novelty and usability of physical activity smart phone applications may help to disseminate information and provide tools to both parents and youth. |
| --- | --- |
| Community organizations and events | Events within the community promoting physical activity have been shown to increase the dissemination of physical activity message [2]. |

## References

1. van Sluijs EM, McMinn AM, Griffin SJ: **Effectiveness of interventions to promote physical activity in children and adolescents: systematic review of controlled trials.** *BMJ* 2007, **335:**703-715.
2. Wong F, Huhman M, Asbury L, Bretthauer-Mueller R, McCarthy S, Londe P, Heitzler C: **VERB™—a social marketing campaign to increase physical activity among youth**. *Preventing Chronic Disease* 2004, **1**.

Craig CL, Bauman A, Gauvin L, Robertson J, Murumets K: **ParticipACTION: A mass media campaign targeting parents of inactive children; knowledge, saliency, and trialing behaviours.** *Int J Behav Nutr Phy* 2009, **6:**88.

Gustafson SL, Rhodes RE: **Parental correlates of physical activity in children and early adolescents.** *Sports Medicine* 2006, **36:**79-97.

1. Rhodes RE, Pfaeffli LA: **Mediators of physical activity behaviour change among adult non-clinical populations: a review update.** *Int J Behav Nutr Phy* 2010, **7:**37.
2. Rhodes RE, Fiala B, Conner M: **A review and meta-analysis of affective judgments and physical activity in adult populations.** *Ann Behav Med* 2009, **38:**180-204.
3. Trudeau F, Shephard RJ: **Physical education, school physical activity, school sports and academic performance.** *Int J Behav Nutr Phy* 2008, **5**.
4. Lubans DR, Foster C, Biddle SJH: **A review of mediators of behavior in interventions to promote physical activity among children and adolescents.** *Preventive Medicine* 2008, **47:**463-470.

Hagger M, Chatzisarantis NLD, Hein V, Soos I, Karsai I, Lintunen T, Leemans S: **Teacher, peer and parent autonomy support in physical education and leisure-time physical activity: A trans-contextual model of motivation in four nations.** *Psychol Health* 2009, **24:**689-711.

1. Marcus BH, Owen N, Forsyth LH, Cavill NA, Fridinger F: **Physical activity interventions using mass media, print media, and information technology.** *American Journal of Preventive Medicine* 1998, **15:**362-378.
2. Marks JT, Campbell MK, Ward DS, Ribisl KM, Wildemuth BM, Symons MJ: **A comparison of Web and print media for physical activity promotion among adolescent girls.** *J Adolesc Health* 2006, **39:**96-104.
3. Shaw G: Canadians tops in social networking. [blogs.vancouversun.com/2009/12/03/canadians-tops-in-social-networking/]
4. LaSalle L: Canadian increasingly migrating to smartphones. [http://www.theglobeandmail.com/news/technology/canadians-increasingly-migrating-to-smartphones/article1245184/]
