# Supplementary material for: Evidence-informed recommendations for constructing and disseminating messages supplementing the new Canadian Physical Activity Guidelines
Source: BMC Public Health. 2013 May 1;13:419. doi: 10.1186/1471-2458-13-419 (PMC3654879; doi:10.1186/1471-2458-13-419)
Supplement: Additional file 6 — Supporting Evidence for the Messaging Recommendations for the New Canadian Physical Activity Guidelines for Adults. This file provides the rationale and lists the supporting evidence for the messaging recommendations for the CPAG for Children. [file 1471-2458-13-419-S6.docx]

# Supporting Evidence for the Messaging Recommendations for the New Canadian Physical Activity Guidelines for Adults

*In text citation numbers correspond with the reference list at the end of Additional File 7

## Target Audience

| Messages should target… | Rationale |
| --- | --- |
| Inactive adults with relatively poor affective attitudes were selected as the primary target group. | Selected a group based on psychological determinants rather than demographic details such as income or gender.  Inactive adults were selected because they have the most to gain [1].  Affective attitudes were selected because they have an empirically supported link to intentions to engage in physical activity [2-4] (. |

## Clarification Messages

Clarification messages should…

- Emphasize that 150 minutes is the minimum for health benefits as opposed to weight loss.
- Clearly describe what is meant by strength versus aerobic training and provide examples of each.
- Clearly describe and provide examples of what is meant by moderate and vigorous intensity activity.
- Convey that physical activity does not have to be traditional gym activities (i.e., running on a treadmill). Clarify how to build in activities that they enjoy.

## Motivational Messages

| Motivational messages should… | Rationale | Example(s) |
| --- | --- | --- |
| Focus on changing affective attitudes for physical activity. | The rationale for starting with attitudes was based in the TPB, where attitudes first influence intentions [4]. | Feel better today! |
| Focus on the enjoyment aspect of physical activity (i.e., how to incorporate physical activity into activities that they enjoy such as spending time with friends). | [4] | Catch up with a friend, take a walk! |
| General form of messages:   1. Address affective attitudes 2. Address self-regulation 3. Close with a call to action, particularly with the emphasis that they can get physical activity in many ways | Changing attitudes may not always lead to increased intentions to be physically activity and furthermore, these intentions may not necessarily lead to behavior change ([5]; intention behavior gap)  Anderson et al. [6] and Rhodes & Pfaeffli [7] provides support for use of self-regulatory skills on PA | Feel better today! Set your goal, enjoy physical activity your way! |

## Channels of Delivery

| Messages should be disseminated through… | Rationale |
| --- | --- |
| Downloadable, electronic tools available at partner websites. Tools to assist with self-regulation should avoid cartoons | Internet is now more common than TV [8] for preferred sources of information and supporting dislike of cartoons. |
| Formerly inactive peers | Watching peers become active can build self-efficacy for PA vicariously [9]. |

## References

1. Warburton DE, Charlesworth S, Ivey A, Nettlefold L, Bredin SS: **A systematic review of the evidence for Canada's Physical Activity Guidelines for Adults.** *Int J Behav Nutr Phys Act* 2010, **7:**39.

# Rhodes RE, Fiala B, Conner M: A review and meta-analysis of affective judgments and physical activity in adult populations. *Ann Behav Med* 2009, 38:180-204.

1. Sheeran P: **Intention-behavior relations: A conceptual and empirical review.** *Exercise and Sport Science Reviews* 2002, **12:**1-36.
2. French DP, Sutton S, Hennings SJ, Mitchell J, Wareham NJ, Griffin S, Hardeman W, Kinmonth AL: **The importance of affective beliefs and attitudes in the theory of planned behavior: Predicting intention to increase physical activity.** *J Appl Soc Psychol* 2005, **35:**1824-1848.
3. Schwarzer R: **Modeling health behavior change: How to predict and modify the adoption and maintenance of health behaviors.** *Appl Psychol- Int Rev* 2008, **57:**1-29.
4. Anderson ES, Wojcik JR, Winett RA, Williams DM: **Social-cognitive determinants of physical activity: The influence of social support, self-efficacy, outcome expectations, and self-regulation among participants in a church-based health promotion study.** *Health Psychol* 2006, **25:**510-520.
5. Rhodes RE, Pfaeffli LA: **Mediators of physical activity behaviour change among adult non-clinical populations: a review update.** *Int J Behav Nutr Phy* 2010, **7:**37.
6. Berry TR, Witcher C, Holt NL, Plotnikoff RC: **A Qualitative Examination of Perceptions of Physical Activity Guidelines and Preferences for Format.** *Health PromotPract* 2008. **11:** 906-916.

# Bandura A: Self-Efficacy - toward a Unifying Theory of Behavioral Change. *Psychol Rev* 1977, 84:191-215.
