# Supplementary material for: Evidence-informed recommendations for constructing and disseminating messages supplementing the new Canadian Physical Activity Guidelines
Source: BMC Public Health. 2013 May 1;13:419. doi: 10.1186/1471-2458-13-419 (PMC3654879; doi:10.1186/1471-2458-13-419)
Supplement: Additional file 7 — Supporting Evidence for the Messaging Recommendations for the New Canadian Physical Activity Guidelines for Older Adults. This file provides the rationale and lists the supporting evidence for the messaging recommendations for the CPAG for Older Adults. [file 1471-2458-13-419-S7.docx]

# Supporting Evidence for the Messaging Recommendations for the New Canadian Physical Activity Guidelines for Older Adults

*In text citation numbers correspond with the reference list at the end of Additional File 8

## Target Audience

| Messages should target… | Rationale |
| --- | --- |
| Apparently healthy older adults aged 65 years and over | Older adults require age-relevant information to motivate them to engage in activity [1,2]. |
| Health professionals | Older adults have reported being influenced by the suggestions, requests, and affirmations of their activity from friends, family and health care workers [3]. |
| Familial caregivers | Familial caregivers often act as gatekeepers for information for older adults, particularly for those who are in poor physical health. |

## Clarification Messages

Clarification messages should…

- Clarify that the recommended levels of physical activity can be done in self-selected blocks. A sample message could state: “150 minutes your way (e.g., 30 minutes 5 days per week, 50 minutes 3 days per week)”
- Provide a “how to” for key pieces of the guidelines that is presented in language that the older adult can understand.
  - Provide clear and concise descriptions of levels of intensity of physical activity.
  - Distinguish between walking and walking for exercise.
  - Provide examples of types of muscle and strength training exercises (e.g., lifting more than you would usually lift, working with resistance bands, lifting weights, doing exercises that use your body weight for resistance, heavy gardening & yard work).
  - Clarify the intensity level required for muscle and strength training exercises. An example clarification messages could state ”Continue each exercise until the point where it's hard to do another repetition without help”.

## Motivational Messages

| Motivational messages should… | Rationale | Example(s) |
| --- | --- | --- |
| Focus on motivating older adults to engage in physical activity of moderate intensity or higher. Aim to make older adults aware that vigorous activity is something of which they are capable | Research has consistently found that moderate to higher intensity physical activity relates to reduced functional limitations and disability in older adults; while evidence for light intensity physical activity is lacking [4]. | Seniors can sweat.  Heavy breathing is not just for the young. |
| Convey that physical activity can be fun and enjoyable. | Messaging campaigns should be grounded in behavioral change theories [5]. Although not specific to older adults, it has been demonstrated that change in affective attitude can be predictive of change in physical activity behavior [6,7]. Moreover, affect is an important source of input in decision making for older adults, especially when mental resources are limited [8,9]. | Enjoy an active life! You’ve earned it!  It may be more fun than you think! |
| Target confidence and concerns about becoming active later in life. Highlight perceived barriers that may not be real barriers, such as age and experience. | Advertising campaigns should be grounded in behavioral change theories [5], as such messages focused on self-efficacy/ perceived behavioral control (i.e., appraisal of confidence/ perception of one’s capability to engage in physical activity). In a recent meta-analysis, interventions aimed at increasing physical activity self-efficacy had a small but significant effect on self-efficacy [10]. | The world can be your gym.  Not/never too late to start! No experience required! |
| Highlight benefits of physical activity that are important to older adults | Maintaining functional independence with age is a greater concern than prevention of disease among older adults [4]. Older adults have also reported that meeting people motivates them to engage in physical activity [11]. | Stay fit. Stay independent. Stay connected |

## Channels of Delivery

| Messages should be disseminated through… | Rationale |
| --- | --- |
| Physicians and other health professionals (e.g., nurses, Physiotherapits, Occupational therapits, Recreation therapists in nursing homes) via the web (CSEP, ParticipACTION, specialty organizations for the each of the health professions) | The web was chosen due to cost constraints. Although older adults may have limited access to information on the web [2,8], the materials could be printed off the web by physicians and other health professionals and then circulated to older adults. |
| The local government (i.e., departments within municipalities that deal with healthy living/seniors) via the web | Messages are more effective when they are from a credible source [12]. The local government plays an essential role in creating a supportive environment for physical activity in older adults [13]. |
| The local government via public relations and other traditional outreach programs/activities | Messages are more effective when they are from a credible source [12]. The local government plays an essential role in creating a supportive environment for physical activity in older adults [13]. |
| Printed brochures distributed to physicians, and health professionals, activity coordinators, or directly to older adults (e.g., community level organizations for seniors, lions clubs, seniors days, physical activity events) | When targeting older adults, printed formats have been recommended over websites [1,2]. If funding allowed, over the long term the messages could be distributed via a printed brochure. |
| Canadian Association of Gerontology | Messages are more effective when they are from a credible source [12]. |

## References

1. Hardy S, Grogan S: **Preventing disability through exercise: Investigating older adults’ influences and motivations to engage in physical activity.** *Journal of Health Psychology* 2009, **14:**1036-146.
2. Berry TR, Witcher C, Holt NL, Plotnikoff RC: **A Qualitative Examination of Perceptions of Physical Activity Guidelines and Preferences for Format.** *Health PromotPract* 2008. **11:** 906-916.
3. Wilson KS, Spink KS: **Exploring older adults' social influences for physical activity**. *Activities, Adaptation & Aging* 2006, **30:** 47-60.
4. Paterson DH, Warburton DE: **Physical activity and functional limitations in older adults: a systematic review related to Canada's Physical Activity Guidelines.** *Int J Behav Nutr Phys Act* 2010, **7:**38.
5. Brawley LR, Latimer AE: **Physical activity guides for Canadians: messaging strategies, realistic expectations for change, and evaluation.** *Appl Physiol Nutr Me* 2007, **32:**S170-S184.
6. Rhodes RE, Fiala B, Conner M: **A review and meta-analysis of affective judgments and physical activity in adult populations.** *Ann Behav Med* 2009, **38:**180-204.
7. Rhodes RE, Pfaeffli LA: **Mediators of physical activity behaviour change among adult non-clinical populations: a review update.** *Int J Behav Nutr Phy* 2010, **7:**37.
8. Peters W: **Age-related changes in decision making.** In *The aging consumer. Perspectives from Psychology and Economics.* Edited by: Drolet A, Schwarz N, Yoon C. New York: Taylor and Francis Group; 2010:75-101
9. Slovic P, Finucane ML, Peters E, MacGregor DG: **The affect heuristic**. In *Heuristics and Biases: The Psychology of Intuitive Judgment.* Edited by: Gilovich T, Griffin D, Kahneman D. New York: Cambridge University Press; 2002:397-420.
10. Ashford S, Edmunds J, French, DP: **What is the best way to change self-efficacy to promote lifestyle and recreational physical activity? A systematic review with meta-analysis.** *British Journal of Health Psychology* 2010, **15:**265–288.
11. Hardy S, Grogan, S: **Preventing disability through exercise : Investigating older adults' influences and motivations to engage in physical activity .** *J Health Psychol* 2009, **14:** 1036-1046.
12. Jones LW, Sinclair RC, Courneya KS: **The effects of source credibility and message framing on exercise intentions, behaviors, and attitudes: An integration of the elaboration likelihood model and prospect theory.** *J Appl Soc Psychol* 2003, **33:**179-196.
13. Dechaine J, Witcher C: Rural Route to Active Aging Focus Group Report: What We Heard in Rural Alberta. [http://www.centre4activeliving.ca/older-adults/rural/focus-report.pdf]
