# Supplementary material for: Evidence-informed recommendations for constructing and disseminating messages supplementing the new Canadian Physical Activity Guidelines
Source: BMC Public Health. 2013 May 1;13:419. doi: 10.1186/1471-2458-13-419 (PMC3654879; doi:10.1186/1471-2458-13-419)
Supplement: Additional file 8: Table S3 — Expert panel. This table lists the names, expertise, and roles of the individuals who participated in the recommendation development meeting. [file 1471-2458-13-419-S8.docx]

# Table 8. Expert panel

| Name | Expertise and Institution | Role(s) |
| --- | --- | --- |
| ***Overarching messages workgroup*** | | |
| Dr. Amy Latimer-Cheung, PhD | Exercise behavior change, physical activity messaging  Assistant Professor, Queen’s University | Co-chair, writing team, CPAG development |
| Dr. Ian Janssen, PhD | Exercise physiology, community health and epidemiology  Associate Professor, Queen’s University | CPAG development |
| Mary Duggan | Manager,CSEP | Key stakeholder, CPAG development |
| Sophie Sommerer | Senior Policy Analyst, Healthy Living Unit Healthy, PHAC | Key stakeholder |
| Sue Ronald | Director, Marketing, Creative Services & E-Comms, PHAC | Key stakeholder |
| Jennifer Tomasone, PhD(c) | Graduate Student, McMaster University | Writing team |
| ***Child workgroup*** | | |
| Dr. Mark Beauchamp, PhD | Exercise behavior change and population health  Assistant Professor, University of British Columbia |  |
| Dr. Mark Tremblay, PhD | Exercise physiology, guideline development  Director, HALO, CHEO Research Institute; Professor, University of Ottawa | Chair of the CPAG Initiative |
| Saskia Jarvis | Policy Analyst, Healthy Communities Division, PHAC | Key stakeholder |
| Marianne Bernardo | Vice President of Marketing, ParticipACTION | Key stakeholder |
| Leanne Maidment | Acting Director, Public Affairs, PHAC | Key stakeholder |
| Gabriella Nasuti, MSc | Project Coordinator, University of Victoria | Writing team |
| ***Youth workgroup*** | | |
| Dr. Guy Faulkner, PhD | Physical activity promotion and messaging among children  Associate Professor, University of Toronto |  |
| Dr. Ryan Rhodes, PhD | Exercise and sedentary behavior change, physical activity messaging  Professor, University of Victoria | Co-chair, writing team, CPAG development |
| Allana LeBlanc, MSc | Researcher Coordinator  Healthy Active Living and Obesity Research Group, CHEO Research Institute | CPAG development  CPAG message development |
| Katherine Janseen | Communications Manager  ParticipACTION | Key stakeholder |
| Heather Gainforth, PhD(c) | Graduate Student, Queen’s University | Writing team |
| ***Adult workgroup*** | | |
| Dr. Tanya Berry, PhD | Exercise behavior change, physical activity messaging  Associate Professor, University of Alberta |  |
| Dr. Kathleen Martin Ginis, PhD | Exercise behavior change, knowledge translation  Professor, McMaster University |  |
| Lori Zehr, MSc | Associate Director, Camosun College | CPAG development |
| Cora Craig, MSc | Canadian Fitness and Lifestyle Research Institute |  |
| Eric Glaude, BJ | Communications Advisor, Public Affairs, PHAC | Key stakeholder |
| Marie-Josée Perrier, PhD(c) | Graduate Student, Queen’s University | Writing team |
| ***Older adult workgroup*** | | |
| Dr. Audrey Hicks, PhD | Neuromuscular physiology and exercise rehabilitation  Professor, McMaster University; President, CSEP | CPAG development |
| Dr. John Spence, PhD | Behavioral medicine, knowledge translation  Professor, University of Alberta |  |
| Dr. Donald H. Paterson, PhD | Donald H Paterson, School of Kinesiology, Canadian Centre for Activity and Aging, University of Western Ontario | CPAG development |
| Patti Fanslau, BA | Advertising & Program Promotion Coordinator Government of Manitoba |  |
| Monica LaBarge, PhD | Marketing communications for health promotion  Assistant Professor, Queen’s University |  |
| Kristina Kowalski, PhD(c) | Research Coordinator & Graduate Student, University of Victoria | Writing team |

CFLRI, Canadian Fitness and Lifestyle Research Institute

CHEO, Children’s Hospital of Eastern Ontario

CSEP, Canadian Society for Exercise Physiology

HALO, Healthy Active Living and Obesity Research Group

PHAC, Public Health Agency of Canada
